# Improving recovery after bowel cancer surgery: mixed methods feasibility study of a co‐produced information intervention (Recover Together)

**DOI:** 10.1111/codi.17210

**Published:** 2024-11-04

**Authors:** Stephen J. Chapman, Sadia Ahmed, Laurie Cave, Kate Morton, James P. Tiernan, Samantha Limbert, Maureen Naylor, Armando Vargas‐Palacios, Maria D. S. Lonsdale, Claire L. Davies, Nikki Rousseau, Deborah D. Stocken, David G. Jayne

**Affiliations:** ^1^ Leeds Institute of Medical Research, University of Leeds Leeds UK; ^2^ Leeds Institute for Clinical Trials Research, University of Leeds Leeds UK; ^3^ Leeds Teaching Hospitals NHS Trust Leeds UK; ^4^ West Yorkshire Ileostomy Association Leeds UK; ^5^ Academic Unit of Health Economics, University of Leeds Leeds UK; ^6^ School of Design, University of Leeds Leeds UK

**Keywords:** colorectal cancer, complex interventions, information, recovery

## Abstract

**Aim:**

Recovery after surgery for colorectal cancer is a complex process, involving numerous physiological, emotional, social and economic challenges. Good information is a key factor for enabling patients to recover well, but there is a paucity of evidence to guide how this should be done. A new information intervention (Recover Together) comprising a booklet, an online video and an inpatient goal board has been developed. This study explores its feasibility, as well as the feasibility of key study methods, during its first use in the United Kingdom National Health Service (NHS).

**Methods:**

This is a mixed methods, multi‐centre, feasibility study of a complex intervention. A total of 105 participants undergoing oncological colorectal surgery will be recruited across three to four study sites in the UK. Participants will receive each component of the Recover Together intervention at defined timepoints before and during hospital admission. A series of patient‐centred outcome instruments will be administered in hospital and during follow‐up at 30 days and 6 months. Outcomes of feasibility will comprise the time taken to establish the intervention at participating sites, assessments of intervention fidelity and acceptability, as well as return rates of key clinical outcome instruments. The mixed methods design will comprise interviews and focus groups with patients and health professionals, non‐participant observation in ward areas and clinics, user‐specific video analytics and daily photographs of the goal boards.

**Discussion:**

The findings of this study will provide a feasibility assessment of the Recover Together intervention when used for the first time in NHS practice. If shown to be feasible, this will guide the development of a future definitive study to explore the clinical and cost effectiveness of the Recover Together intervention to improve recovery after surgery.

**Clinical Trials Registration:**

ISRCTN62430915.

**What does this paper add to the literature?**

Patient counselling prior to major oncological bowel surgery is an important aspect of enhanced recovery. However, little evidence exists on how best to deliver this effectively. This protocol describes a rigorous process for evaluating the feasibility and plausibility of a novel information intervention, deployed for the first time in the UK National Health Service.

## INTRODUCTION

Bowel cancer is the fourth most common cancer in the UK, with approximately 42 000 new cases reported each year. In over 60% of patients, the treatment involves major surgery followed by a lengthy period of recovery [1]. This is a complex process involving a myriad of physiological, emotional, social and economic factors which define a patient's post‐treatment quality of life. Recovery begins before surgery whilst patients and their families prepare for hospital and continues for several months after discharge [2].

Enhanced recovery after surgery guidelines were first introduced in the 1990s and are the standard of care in many healthcare systems throughout the world [3]. Patient counselling is a fundamental component of these guidelines, essential for improving patients' understanding of recovery, improving their experience of treatment, and reducing complications. Whilst current guidelines provide a ‘strong’ recommendation for counselling and providing information to patients about their recovery, current approaches are based on sparse evidence. In a previous systematic review, studies of information and education initiatives around the time of colorectal surgery reported mixed signals of patient benefit across a heterogeneous selection of clinical outcomes [4]. In a qualitative study exploring patients' perspectives of information initiatives, concerns were raised that existing approaches are impractical and challenging to navigate during highly anxious circumstances [5]. These studies made clear that higher quality evidence is required to guide this important aspect of care.

To address this unmet need, we previously co‐developed a multi‐modal information intervention called ‘Recover Together’. This was an iterative process guided by the National Institute for Health and Care Research and Medical Research Council framework for developing complex interventions and was developed collaboratively between patients, clinicians and experts in information and communication design [6]. The process considered key areas of patient need identified from earlier work as well as co‐design workshops to develop, adapt, prototype and finalise each component of the intervention. These comprised a written booklet, a motion video and an ‘end‐of‐the‐bed’ goal board [7].

Previous studies exploring new information initiatives relevant to colorectal surgery are limited by a lack of evidence for their promise and plausibility. The clinical outcomes reported in these studies are also heterogeneous, often lacking considerations of patient‐centred benefit. The aim of this study is to explore the feasibility of the Recover Together intervention when deployed for the first time in the UK National Health Service (NHS). It also aims to explore the feasibility of key methods, including return rates for a series of patient‐centred outcome instruments and the return of data required to generate a robust evaluation of cost effectiveness in a future study.

## METHODS

### Ethics and governance

Ethical approval for this study was obtained from the West of Scotland Research Ethics Service (22/WS/0136) on 31 August 2023. It was registered prospectively on the ISRCTN registry (ISRCTN62430915) on 10 October 2023 and began enrolling participants on 19 December 2023. The sponsor of the study is the University of Leeds and oversight is provided by a dedicated study management group. The present paper is reported in line with the Standard Protocol Items: Recommendations for Interventional Trials 2013 Checklist [8].

### Aims and objectives

The study aims to explore the feasibility of the Recover Together intervention when deployed in the UK NHS, including to

- Estimate the timescale for setting up the intervention at clinical sites
- Explore fidelity across each resource, reasons and the impact of any adaptation
- Explore user (patient/staff) acceptability of the resources over time and across settings
- Explore user (patient/staff) engagement with the resources over time and across settings
- Refine training/implementation materials for use during a definitive trial.

The study also aims to address methodological uncertainties to inform the design of a future definitive trial, including to

- Explore completion rates of a core series of patient‐centred outcome instruments [9]
- Estimate rates of missing data relating to costs/resource utilisation and to finalise a health‐economic data collection instrument for use during a definitive trial.

### Study design

**FIGURE 1:**
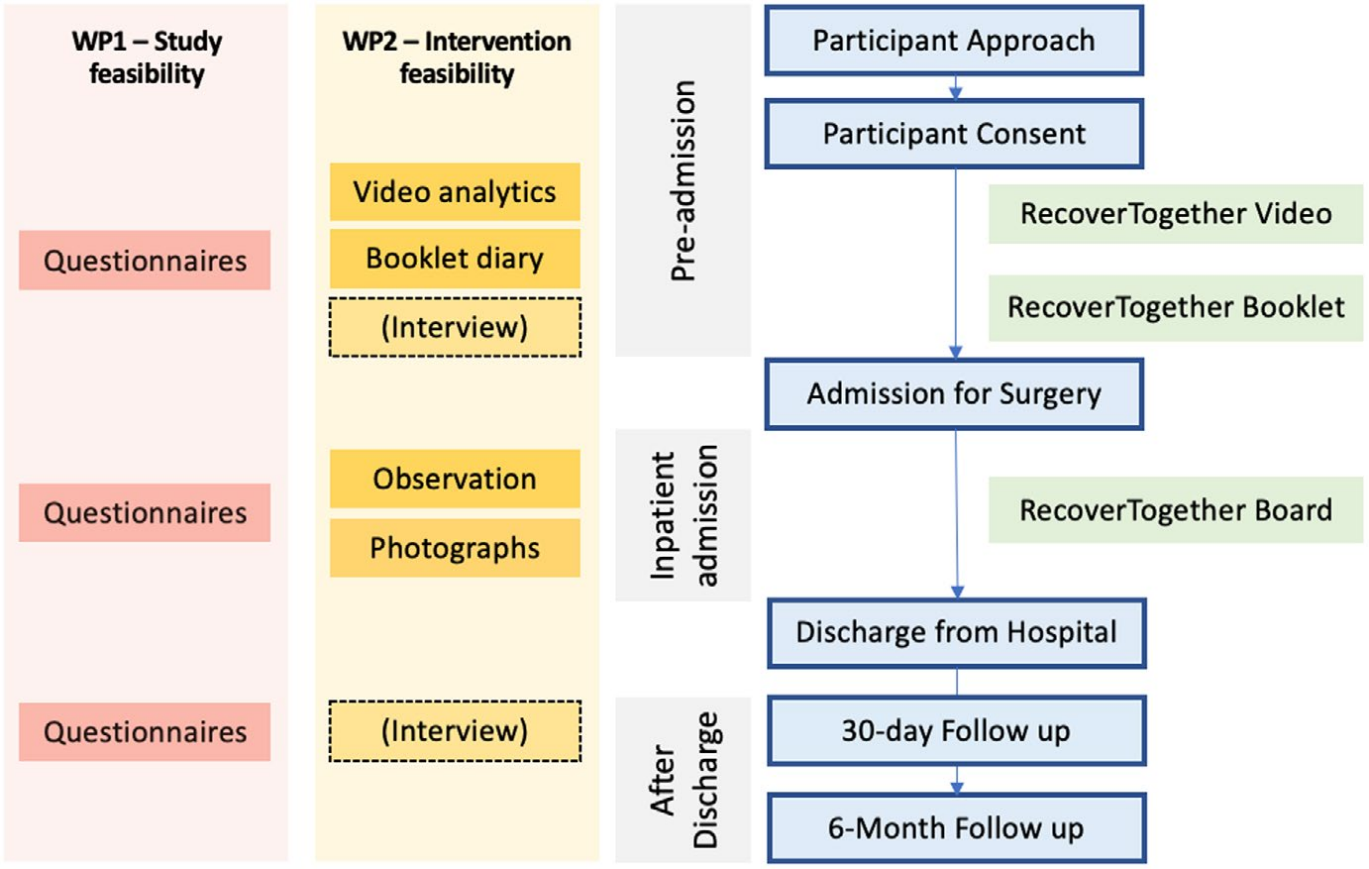
Study schema.

This study is a mixed methods, feasibility study of a complex intervention developed for the purpose of improving recovery after oncological colorectal surgery. A total of 105 participants will be recruited from outpatient clinics across three to four study sites in the UK. After providing written informed consent, participants will receive each component of the Recover Together intervention at various timepoints, including around the time of their preoperative counselling and during their inpatient hospital admission. Participants will be followed up at 30 day and 6 month timepoints (Figure 1).

### Study setting

A total of three to four secondary‐ or tertiary‐care hospitals will join the study. Hospitals who have begun recruiting to the study are outlined in Supplement S1. They represent a range of NHS structural organisations and patient volumes. The geographical selection of sites will assist us in reaching a diverse and representative population.

### Eligibility criteria

To be eligible for the study, patients must be aged ≥18 years, able to provide written informed consent, and plan to undergo elective colorectal surgery (with or without a stoma) for the indication of confirmed or suspected colorectal cancer. No further exclusion criteria will apply, including those relating to literacy or disability. Recruitment materials will be available in Urdu (British) to explore potential challenges of recruitment in non‐English languages. Urdu was chosen based on discussion with participating sites regarding which additional language would have most impact on study accessibility within their communities.

### Intervention

**FIGURE 2:**
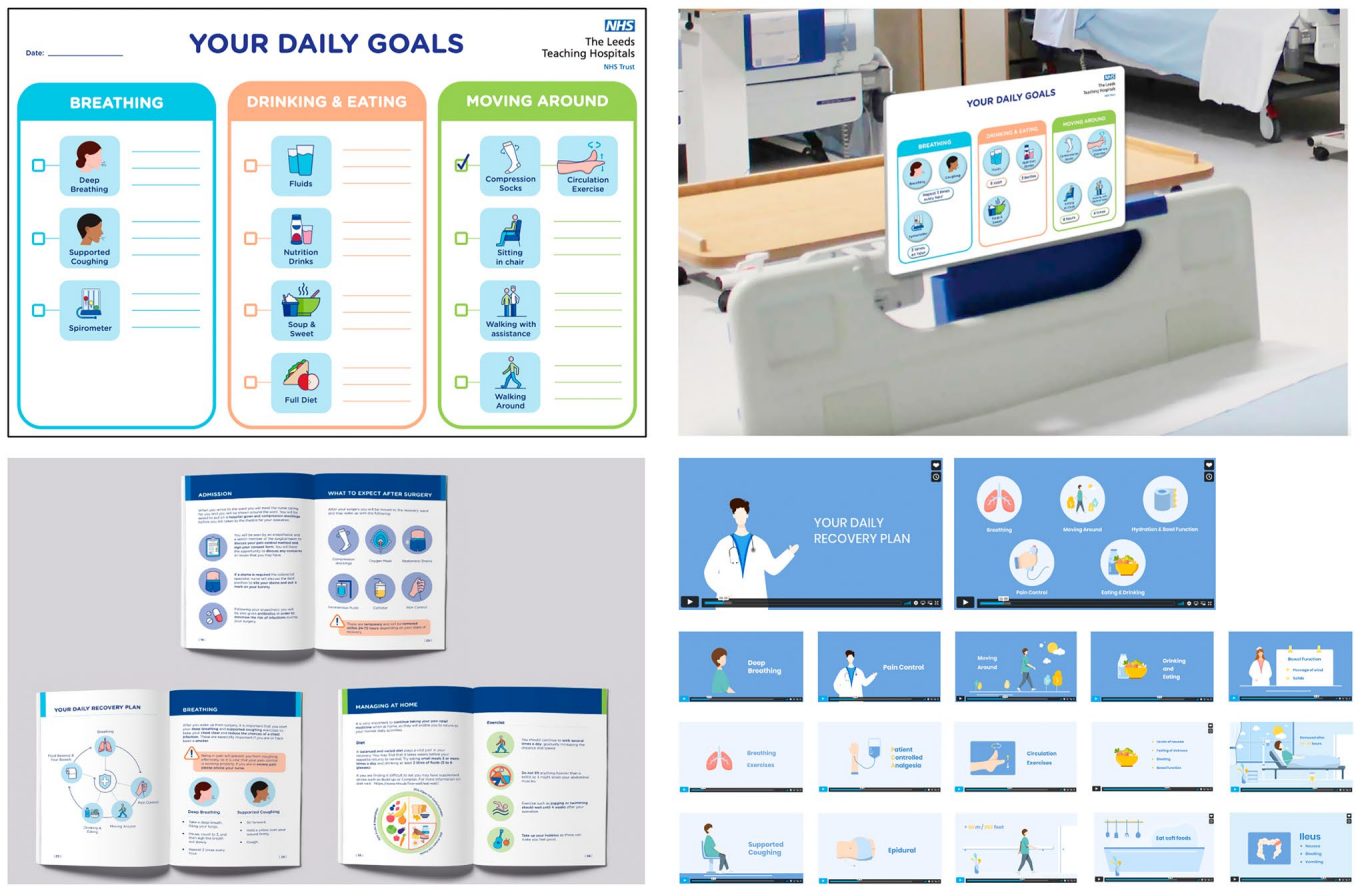
Recover Together intervention components. Top row: Recover Together goal board (left, patient‐facing view; right, schematic impression in situ); bottom left; Recover Together booklet; bottom right: Recover Together video (storyboard).

A multi‐modal information intervention (Recover Together) was co‐developed by patients, healthcare staff and experts in the field of information and communication design during our earlier work. The resulting intervention incorporated stakeholder priorities as well as design and cognitive principles [7]. A further co‐development process was undertaken to adapt the intervention for Urdu speakers. All resources are available in English and Urdu languages, with bilingualists able to choose from either version. The components comprise the following (Figure 2).

- Recover Together video: an online, narrated, motion video to provide patients and their families with an overview of surgery and their recovery. It is administered to participants before hospital admission and used at home and/or in hospital.
- Recover Together booklet: a full‐colour, illustrated, plain English/Urdu, A5 glossy booklet used to guide patients through each stage of recovery. Major sections include Preparing for Surgery; Day of Surgery; Recovery in Hospital; and Recovery at Home. It is administered to participants before hospital admission and used at home and/or in hospital.
- Recover Together goal board: a patient‐facing, ‘end‐of‐the‐bed’, whiteboard for daily goal‐setting between patients and clinicians. This aims to encourage engagement and shared decision‐making across key domains of recovery (breathing, nutrition, mobility). It is administered to participants after surgery and used during their inpatient stay.

**FIGURE 3:**
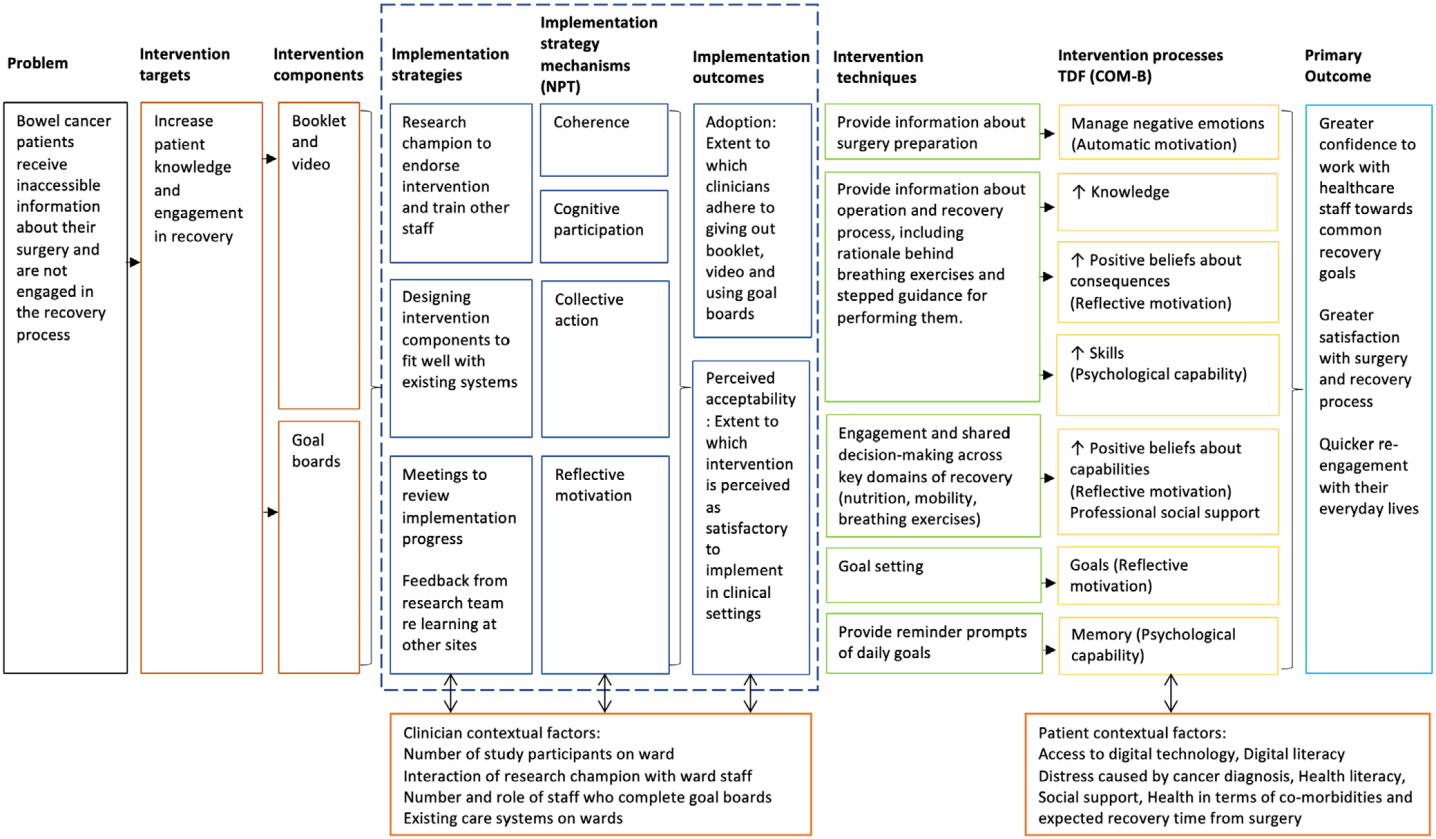
Recover Together intervention logic model.

The intervention is designed to give patients greater confidence to work with healthcare professionals towards common recovery goals. The theorised mechanisms by which this will be achieved are outlined in the logic model (Figure 3). This draws on the Theoretical Domains Framework, exploring behaviours and issues relating to the implementation of new healthcare interventions [10]. Mechanisms relating to the booklet and video comprise managing negative emotions, increasing knowledge about recovery, enhancing positive beliefs around consequences, and increasing skills (i.e., psychological capability). Expected mechanisms for the goal board comprise increasing positive beliefs around capabilities, setting goals and enhancing memory. The ‘core processes’ outlined in Table 1 describe how each component of the Recover Together intervention will be delivered if implemented as planned.

**TABLE 1:** Core processes for intervention delivery and usage.

Intervention component	Criteria	How collected	Definition of per cent adherence per resource	Definition of per cent adherence per participant
Video	Intervention delivery: an explanation of the video and access instructions provided by the healthcare professional during a pre‐admission appointment or via post/telephone	Baseline CRF	Across all sites, what percentage of patients recruited to the study receive an explanation of the video, according to the baseline CRF?	What percentage of the six core processes does each participant meet?
	Intervention usage: patients view the video at least once before or after their operation	Vimeo	Across all sites, what percentage of patients recruited to the study view the video at least once at any timepoint during the study?	
Booklet	Intervention delivery: patients receive the booklet during a pre‐admission appointment or via post	Baseline CRF	Across all sites, what percentage of patients receive the booklet during their pre‐admission appointment or are sent it via post, according to the baseline CRF?	
	Intervention usage: the patient uses the booklet at least once before, during or after hospital admission	Self‐report patient diary	Across all sites, what percentage of patients report using the booklet at least once, whether that be before, during or after hospital admission?	
Goal board	Intervention delivery: an explanation of the goal board is provided by the healthcare professional during a pre‐admission appointment or via post/telephone	Baseline CRF	Across all sites, what percentage of patients receive an explanation of the goal board before their operation, according to the baseline CRF?	
	Intervention usage: goal board is used and updated at least once	Photos	Across all sites, what percentage of patients are provided with the goal board and receive at least one update to it during their hospital stay?	

Abbreviation: CRF ‐ Case Report Form

### Study outcomes

A series of patient‐centred outcomes and respective endpoints will be assessed, as recommended by the Core Outcome Measures for Perioperative and Anaesthetic Care‐Standardised Endpoints in Perioperative Medicine (COMPAC‐StEP) initiative [9]. This is an agreed set of outcomes agreed by clinicians for use in studies of perioperative medicine. A full outline of instruments and timings of administration is described in Table 2.

**TABLE 2:** Agreed outcome instruments.

Instrument	Outcome domain	Study timepoint(s)	Method of administration
Bauer Patient Satisfaction Questionnaire	Patient satisfaction	Day 1 after surgery	Administered by researcher/research delivery team on inpatient ward
Quality of Recovery 15 (QoR‐15)	Patient well‐being	Baseline Day 3 after surgery	Administered by researcher/research delivery team in clinic (baseline) and ward (after surgery)
EQ‐5D‐5L	Health‐related quality of life	Baseline 30 days after surgery 6 months after surgery	Administered by researcher or research delivery team in clinic (baseline) and via post/phone/clinic (after surgery)
WHO Disability Assessment Schedule	Functional status	Baseline 6 months after surgery	Administered by researcher or research delivery team in clinic (baseline) and via post/phone/clinic (after surgery)
Resource‐use questionnaire	Resource use	Baseline 6 months after surgery	Administered by researcher or research delivery team in clinic (baseline) and via post/phone /clinic (after surgery)

Abbreviations: EQ‐5D‐5L, EuroQol‐5 Dimensions‐5 Levels; WHO, World Health Organisation.

### Quantitative data collection methods

A series of quantitative methods will be used to explore fidelity and compliance with each of the Recover Together components according to the ‘core processes’. These will include the following.

- Health‐professional‐reported administration of the intervention—Using case report forms, healthcare professionals will be asked to self‐report whether they administered and counselled participants on each component, as well as reasons if this did not occur.
- Patient self‐reported use of the Recover Together booklet—Using a feedback form integrated into the booklet, patients will self‐report which sections of the booklet they looked at, and whether this was before, during or after their hospital admission. An open‐text section will collect comments on what they liked or disliked about the booklet.
- User‐specific analytics of the Recover Together video—Analytics (view frequency, timing, duration and device platform) will be recorded using the Vimeo hosting platform (Vimeo Inc., NY, USA). Participants will receive a unique access link, providing the research team with detailed insights into user‐specific patterns of video usage.
- Photos of the Recover Together goal board—Daily photos of the board will be taken to record daily patterns of use, as well as to quantify the frequency with which goals change and the extent to which they are individualised.

A full list of data items relating to the confirmation of eligibility, baseline demographics and treatment characteristics is provided in Supplement S1.

### Qualitative data collection methods

Qualitative methods will be used to explore acceptability of the intervention amongst patients and health professionals. They will also provide insights around the mechanisms of impact (such as empowerment and confidence). An outline of these methods is summarised below.

- Interviews with patients—These will take place before and up to 6 months after surgery, capturing experiences at different stages of recovery. Some patients will take part in a single interview after surgery (*n* = 12–15), whilst others will take part in two interviews, including one before and one after surgery (*n* = 9–12). This will enable an interval assessment of how perspectives change over time. Sampling will be purposive, informed by factors theorised to influence engagement, including age, sex, ethnicity, type of surgery, chemotherapy and preoperative pathway to surgery, and comorbidities. Interviews will be semi‐structured and will take place in person or remotely for 30–60 min. Participants will be permitted to invite a relative or other co‐participant if they wish. A short survey administered after the interview will explore participants' health literacy [11].
- Non‐participant observation—Non‐clinical researchers will observe the goal board in use (up to 24 sessions), including the involvement of patients and healthcare staff in setting goals. The timing of sessions will be spread across the study period and may be informed by photos of the goal board (i.e., a session may be prompted by a change in pattern of use). Field notes will be taken comprising detailed descriptions of observations, comments on theories about how the resources are being used, and subjective reflections.
- Interviews with healthcare staff—Surgeons, ward nurses, clinical support workers and nurse specialists (*n* = 15–18) will take part in a single interview to explore barriers to implementing the intervention resources, reasons for adaptations and their subsequent impact, and unintended consequences. Sampling will be purposive, informed by participants' healthcare role, level of seniority, number of patients who have been recruited to the study from their ward, and their level of engagement with the intervention. Staff with experience of introducing the video and booklet in clinic as well as those with experience of administering the goal board on the ward will be approached. Interviews will be semi‐structured and will take place in person or remotely.
- Focus groups with extended healthcare staff—One focus group will be facilitated at each study site (total *n* = 3–4) to explore perspectives of the wider team regarding barriers to implementation, adaptations and unintended consequences of the intervention resources. Participants will include non‐clinical ward staff and allied professions such as dietitians and physiotherapists. A total of six to eight participants will take part in each group at the end of the recruitment period. Groups will be held in person or remotely and will last approximately 60 min, followed by a short survey about their role and experience with the intervention.

### Progression criteria

Decisions around the feasibility of the intervention and study methods will be guided by a series of progression criteria. The decision to progress to a definitive study will be determined according to a traffic light assessment for each feasibility outcome (red, not feasible; amber, probably feasible with modification; green, feasible without modification) (Table 3). Amber outcomes will be considered using the ADepT framework, a systematic approach to decision‐making involving the identification, appraisal and agreement of changes to the design of an intervention or study method [12].

**TABLE 3:** Feasibility progression criteria.

Criteria	Progress (feasible without change)	Amber (probably feasible with modification)	Stop (not feasible)
Intervention feasibility (Work Package 1)			
Time taken for intervention set‐up across participating sites	Time to first patient approach <3 months after HRA approval	Time to first patient approach 3–6 months after HRA approval	Time to first patient approach >6 months after HRA approval
Compliance to core processes per resource	>75%	50%–75%	<50%
Compliance to all core processes per participant	>75%	50%–75%	<50%
Study method feasibility (Work Package 2)			
Return rate per measurement instrument	>75%	50%–75%	<50%
Return rate of all instruments per participant	>75%	50%–75%	<50%
Cost/resource utilisation missing data	>75%	50%–75%	<50%

Abbreviation: HRA ‐ Health Research Authority.

### Statistical and qualitative analysis

Quantitative data will be presented descriptively as rates (categorical) and means (continuous). No formal statistical comparisons or hypothesis testing will be conducted. Data will be compared with existing published literature to explore possible signals of patient benefit.

Rapid qualitative analysis will be used to enable important findings emerging from interviews and observations to be identified during the data collection phase in order to inform ongoing optimisations to the feasibility study [13]. Based on initial data collection, a Rapid Analysis Protocol sheet will be developed which will subsequently be used to summarise key data for each interview/observation, as relevant to the developing analysis. Regular meetings of the qualitative team will take place throughout the data collection period to discuss the developing analysis and to make decisions about further data collection. Analysis will focus on understanding how the intervention was used and perceived at different points in time (pre‐surgery, whilst in hospital, shortly after coming home and after being at home for a while) and in different settings (the various hospital sites). To avoid losing important contextual details about the timepoint and setting in which each person's data were collected, a pen portrait analysis will be undertaken which draws out the narrative of how the intervention was used over time [14]. This involves identifying the core focus of the analysis (i.e., probably relating to acceptability and engagement with the intervention over time and across sites and how it was adapted). A template will be devised for capturing narrative summaries of the factors influencing acceptability, engagement and adaptations for staff and patients at each site. This will enable data from the interviews and observations to be pulled together to generate a rich understanding of the overall process in a way that facilitates understanding of similarities and differences between sites. Once a pen portrait has been generated for each site, an interpretation phase will occur in which the research team seek to explain why patients and staff engaged over time in the way that they did. These conceptual insights will be developed inductively, but may subsequently be mapped to Normalisation Process Theory [15] and the Theoretical Framework of Acceptability [16] to build our understanding. During the process of developing the pen portraits, we will hold a co‐analysis workshop in which provisional portraits will be reviewed and interpreted by a group of patient representatives.

Following an initial analysis of the qualitative and quantitative data, to obtain additional insights we will conduct a ‘following a thread’ mixed methods analysis. This involves generating questions raised by the results of one component and following ‘the thread’ across to the other component(s), for example looking for explanations in the qualitative data for patterns observed in the quantitative data.

### Intervention refinement

Co‐design stakeholder workshops will be organised with surgeons, nurses and patients (two workshops with *n* = 9 participants) to refine the intervention (if required) and to refine training/implementation materials and processes. The first workshop will focus on identifying essential refinements based on qualitative and quantitative feasibility findings, and the second workshop will focus on ratification of the materials in preparation for the next stage of evaluation. The ratified training/implementation materials will feed directly into the future evaluation as an essential resource during site set‐up.

### Public and community involvement

A patient and public advisory panel has been convened to work closely with the study team throughout the study, including advising on data collection and recruitment processes, contributing to plain language versions of the study findings, developing recruitment materials, developing topic guides, interpreting data, and dissemination and engagement activities. In particular, the advisory panel will participate in a co‐analysis session to assist in the interpretation of the qualitative data and to inform the mixed methods analysis. A community group of members of the public who speak Urdu has also been convened. They will work with the study team to ensure that Urdu adapted materials are developed appropriately and further adapted based on feedback from the feasibility results.

## DISCUSSION

This study will explore the feasibility of implementing and evaluating the Recover Together intervention in NHS surgical practice. A series of quantitative and qualitative methods will explore the fidelity of each intervention component (booklet, video, goal board), reasons for adaptations and the impact of these, as well as acceptability of the components amongst patients and health professionals. The study will also explore the feasibility of administering a series of patient‐centred outcome instruments along with strategies to mitigate burden. Overall, this will refine the development of a future definitive study to explore the clinical and cost effectiveness of the Recover Together intervention, if shown to be feasible.

Patient counselling is a fundamental aspect of fast‐track recovery but little evidence exists to inform how it should be done in practice [4]. This is important so that pat**i**ents are enabled to participate actively in their recovery and to re‐engage with their everyday lives [17]. The Recover Together intervention was co‐produced by patients, health professionals and communication design experts to address common challenges identified in our previous engagement work. These included considerations around emotional stress precluding good understanding; poor information design; a lack of personalised content; missed opportunities to empower patients during their recovery; and poor availability of information after discharge [4]. Possible benefits for patients of addressing these challenges were shown to be an enhanced situated understanding of their care (i.e., familiarisation with the environment, events and people around them) and greater confidence to work with health professionals towards recovery goals [18]. Possible benefits for the NHS are reduced costs, reduced bed days, and greater capacity to provide cancer services in the setting of rapidly increasing demand.

Some issues of feasibility are beyond the scope of this work. First, the study is not designed to explore the feasibility of recruitment and retention, although participant screening data will provide some information about this. Instead, it is likely that recruitment and retention (including strategies to overcome hurdles) will be explored within an internal pilot phase of a potential definitive study. Secondly, the study is not designed to explore the feasibility of randomisation in a future study. Key considerations for a future trial will be the unit of randomisation (i.e., participants or clusters) along with statistical estimates of sample size and/or the intra‐cluster correlation coefficient. Finally, although the study will explore return rates of a series of patient‐centred outcome instruments, this will not determine the choice of a primary outcome. Instead, this will be determined between public representatives and the study management group in consultation with other key stakeholders to ensure that the final results are relevant and important to patients and the health service.

## STUDY SPONSOR

University of Leeds.

## PROTOCOL VERSION

v1.1.

## DATA MONITORING

As a feasibility study of a low‐risk intervention, a data monitoring committee has not been recruited for the purpose of this study.

## DISSEMINATION

The final results will be disseminated via academic conferences, learned publications and social media channels.

## AUTHOR CONTRIBUTIONS

**Chapman S J:** Conceptualization; investigation; funding acquisition; writing – original draft; methodology; project administration; formal analysis; data curation. **Sadia Ahmed:** Investigation; methodology; writing – review and editing; formal analysis; data curation. **Laurie Cave:** Investigation; methodology; writing – review and editing; formal analysis; data curation. **Kate Morton:** Investigation; methodology; writing – review and editing; data curation. **James P. Tiernan:** Writing – review and editing; investigation; funding acquisition. **Samantha Limbert:** Investigation; writing – review and editing; funding acquisition. **Maureen Naylor:** Investigation; funding acquisition; writing – review and editing. **Armando Vargas‐Palacios:** Investigation; funding acquisition; methodology; writing – review and editing. **Maria D. S. Lonsdale:** Investigation; funding acquisition; writing – review and editing; resources; supervision. **Claire L. Davies:** Methodology; investigation; writing – review and editing. **Nikki Rousseau:** Investigation; funding acquisition; methodology; writing – review and editing; formal analysis; supervision. **Deborah D. Stocken:** Writing – review and editing; methodology; supervision. **David G. Jayne:** Conceptualization; funding acquisition; writing – review and editing; supervision.

## FUNDING INFORMATION

This project is funded by the National Institute for Health and Care Research (NIHR) under its Research for Patient Benefit (RfPB) Programme (Grant Reference Number NIHR203660). The views expressed are those of the author(s) and not necessarily those of the NIHR or the Department of Health and Social Care.

## CONFLICT OF INTEREST STATEMENT

The authors have no conflict of interest to declare.

## Supporting information


**Data S1:** Supporting information.

## Data Availability

Data sharing is not applicable to this article as no new data were created or analyzed in this study.
