# Supplementary material for: Improving recovery after bowel cancer surgery: mixed methods feasibility study of a co‐produced information intervention (Recover Together)
Source: Colorectal Dis. 2024 Nov 4;27(1):e17210. doi: 10.1111/codi.17210 (PMC11683172; doi:10.1111/codi.17210)
Supplement: Supplementary file 1 — Data S1: Supporting information. [file CODI-27-0-s001.docx]

**Improving recovery after bowel cancer surgery: Mixed-method feasibility study of a co-produced information intervention (Recover Together)**

**SUPPLEMENT S1**

### *Study Setting*

- Site 1: University-affiliated teaching hospital, NHS Trust. A total of 257 bowel cancer operations were performed in 2018-19 according to the National Bowel Cancer Audit (23)
- Site 2: District general hospital, NHS Foundation Trust. A total of 147 bowel cancer operations were performed in 2018-19 (23)
- Site 3: District general hospital; NHS foundation Trust. A total of 74 bowel cancer operations were performed in 2018-19 (23)
- Site 4: University-affiliated teaching hospital; NHS foundation Trust. A total of 62 bowel cancer operations were performed in 2018-19 (23)

*Data Collection*

Eligibility and baseline data collection - Participants will be screened for eligibility according to the eligibility criteria. After consent, data will be collected including (but not limited to):

- Demographics such as age, sex, ethnicity, body mass index (height and weight), languages people speak and read, American Society of Anesthesiologists grade, and smoking status.
- Clinical co-morbidities, including diabetes mellitus, cardiorespiratory disease, gastrointestinal disease, renal disease, and previous abdominal surgery.
- Details of pre-treatment investigations, including diagnosis, blood parameters and details of pre-admission treatment (chemo/radiotherapy).

Operative and postoperative data will be collected on the day of surgery and during admission. Data will be collected including (but not limited to):

- Operative characteristics: procedure, operative approach (laparoscopic, robotic, laparoscopic), need for conversion from laparoscopic/robotic to open, formation of stoma
- Recovery characteristics: time to gastrointestinal recovery, and time to first mobilisation, nutrition interventions, perioperative line intervention.
